# Low titers of measles antibody in mothers whose infants suffered from measles before eligible age for measles vaccination

**DOI:** 10.1186/1743-422X-7-87

**Published:** 2010-05-06

**Authors:** Hong Zhao, Pei-Shan Lu, Yali Hu, Qiaozhen Wu, Wenhu Yao, Yi-Hua Zhou

**Affiliations:** 1 Department of Infectious Diseases, Nanjing Second Hospital, Nanjing, China; 2 Department of Expanded Program on Immunization, Jiangsu Provincial Center for Disease Control and Prevention, Nanjing, China; 3 Department of Obstetrics and Gynecology, Nanjing Drum Tower Hospital, Nanjing University Medical School, Nanjing, China; 4 Departments of Laboratory Medicine and Infectious Diseases, Nanjing Drum Tower Hospital, Nanjing University Medical School, Nanjing, China; 5 Jiangsu Key Laboratory for Molecular Medicine, Nanjing University Medical School, Nanjing, China

## Abstract

**Background:**

Resurgence or outbreak of measles recently occurred in both developed and developing countries despite long-standing widespread use of measles vaccine. Measles incidence in China has increased since 2002, particularly in infants and in persons ≥ 15 years of age. It is speculated that infants may acquire fewer measles IgG from their mothers, resulting in the reduced duration of protection during their early months of life. This study aimed to clarify the reason of increased susceptibility to measles in young infants in China. Measles IgG in 24 measles infants ≤ 9 months of age and their vaccinated mothers was quantitatively measured. The mean measles neutralizing titer in the vaccinated mothers and in 13 age-match women with the histories of clinical measles were compared.

**Results:**

All the mothers were confirmed to be vaccinated successfully by the presence of measles IgG. Six vaccinated mothers were positive for measles IgM and had high concentrations of measles IgG and the neutralizing antibody, indicating underwent natural boosting. The mean measles neutralizing titer in 18 vaccinated mothers without natural boosting were significantly lower than that in 13 age-match women with the histories of clinical measles (1:37 vs 1:182, *P *< 0.05).

**Conclusions:**

Our results suggest that infants born to mothers who acquired immunity to measles by vaccination may get a relatively small amount of measles antibody, resulting in loss of the immunity to measles before the vaccination age. Measures to improve the immunity in young infants not eligible for measles vaccination would be critical to interrupt the measles transmission in China.

## Background

Measles vaccine was introduced in China in 1965. Since early 1980s, China has implemented a routine two-dose vaccine schedule in children, with the first vaccinated at 8 months of age and the second given at 7 years old (shift to 18-24 months of age since 2006). Reported measles cases in China substantially reduced; the annual incidence per 100 000 population dropped from 200-1500 cases before the vaccine era to 5.7 cases between 1996 and 2000 [1]. However, during recent years, the annually measles cases significantly increased. The reported cases rose from 74813 in 2003 and 71013 in 2004 to 124,219 in 2005 and 100,267 in 2006, with the incidences of 9.56 and 7.67 per 100 000 population respectively [2]. Moreover, the age distribution of measles has changed markedly. In 1990s, 1.74% of reported cases occurred among children < 8 months of age [3]; however, of cases reported during 2003 and 2004, 4.31% and 2.25% occurred among children < 8 months of age respectively, and during 2005 and 2006, the proportion of cases occurring in children < 8 months old increased to 7.62% and 10.98% respectively [2]. In a hospital in Shanghai, nearly 60% of 503 hospitalized children with measles in 2005 were younger than 9 months old [4]. These data demonstrate that young infants in China have increased susceptibility to measles virus.

Although low measles vaccine coverage was putatively considered to be the main cause of the increased measles cases in China [4,5], a nationwide survey in 2004 showed that the measles IgG seroprevalence was as high as 92.9% in children aged 1 to 12 years [6], even slightly higher than the prevalence of some 90% in 1990s [3], during which the measles incidence was lowest. Women who recovered from measles usually have high measles antibody levels whereas those who did not have measles but were successfully vaccinated have relatively lower antibody titers [7]. Currently, the measles antibodies in the vast majority of mothers in China are induced by vaccination rather than by the natural infection. Therefore, it is speculated that infants may acquire fewer antibodies from their mothers, resulting in the reduced duration of protection against measles and leading to the onset of measles before the age of receiving vaccine. In this study, we quantitatively measured the measles IgG in serum samples collected from ill infants in the acute phase and compared the neutralizing measles antibody in mothers of the ill infants and in age-matched women who recovered from measles. We found that mothers who were vaccinated with measles vaccine had significantly lower measles antibody than the age-matched women who had the histories of measles in their childhood. Our data suggest that infants born to the vaccinated mothers may get a relatively small amount of maternal measles antibody, resulting in loss of the immunity to measles before the vaccination age. Thus, measures to improve the immunity in young infants not eligible for measles vaccination would be critical to interrupt the measles transmission in China.

## Results and Discussion

Twenty-four ill infants aged ≤ 9 months old, hospitalized from March 16 to July 16, 2007, and their 24 mothers were enrolled in the study. Each clinically diagnosed case was confirmed by positive measles IgM antibody. The general characteristics of the ill infants and their mothers are shown in Table 1. Five infants received the measles vaccine 2-12 days before onset of the disease. The mean time between blood sampling and onset of the fever was 6.2 days. All infants recovered and were discharged within 3 weeks. All the mothers had the histories of measles vaccination but none of them had a history of clinically and/or serologically confirmed measles, and had any symptom when the blood samples were taken. The mean age of the mothers was 28.3 years (24 to 32), comparable to the mean age of 29.5 years (25 to 34) of 13 women who had histories of clinically diagnosed measles.

**Table 1:** Measles antibodies in sera from ill infants and their mothers

Pair No	Mother			Infant^c^				
		
	IgG (U/ml)	NT^a^	MV IgM^b^	Age (month)	Sex	Day after onset of fever	IgG (U/ml)	Vaccine^d^
1	1272	12	-	3	M	5	49	-
2	42986	256	0.621	8	F	5	80	-
3	77355	>2048	0.302	5	M	7	97	-
4	1782	8	-	7	M	8	54	-
5	3735	32	-	8	M	8	82	-
6	75000	2048	0.552	8	M	7	39	-
7	7069	16	-	8	F	7	42	-
8	2577	32	-	7	M	6	53	-
9	75000	>2048	0.577	5	M	6	59	-
10	275	4	-	9	F	9	94	10
11	5261	24	-	7.5	M	4	73	-
12	55843	1024	0.489	9	M	8	110	6
13	5158	96	-	8	F	6	64	-
14	1391	16	-	8	M	6	52	-
15	23078	192	-	7	M	5	61	-
16	3398	24	-	8	M	3	72	8
17	4849	24	-	8	F	7	54	-
18	3205	16	-	8	F	6	428	12
19	2140	16	-	8.5	F	9	168	2
20	19002	>2048	0.376	6	M	5	50	-
21	2147	48	-	6	M	4	44	-
22	588	12	-	8	M	6	43	-
23	5433	32	-	9	M	5	45	-
24	11963	64	-	9	M	6	70	-

^a ^NT, neutralizing titer.

^b ^The assay's OD450 cut-off is 0.262; -, lower than 0.262.

^c ^All infants were positive for measles IgM.

^d ^-, not vaccinated; number indicates the days after measles vaccination.

All 24 mothers had detectable measles IgG antibody as shown in Table 1. With the known vaccination histories in these mothers, the results validate that all the mothers had been successfully vaccinated. Surprisingly, some of them had extremely high antibody titers. This had us suspect whether these mothers were recently exposed to the wild type virus because they took care of their ill infants, resulting in anamnestic responses with a rapid rise of IgG antibody known as natural boosting. Thus, we tested measles IgM in all 24 mothers; 6 of them were positive with the OD450 values ranging from 0.302-0.621 (Table 1). Infection of measles virus in persons with the history of vaccination may have detectable IgM [8,9]. Compared with the high concentrations of measles IgG in their sera (Table 1), the relatively low OD450 values (0.302-0.621) of the specific IgM in the ELISA test strongly suggest that these 6 mothers were exposed to the virus in the presence of pre-existing measles IgG, rather than the primary infection in the absence of immunity, because the ratio of IgM to IgG after re-exposure, if IgM is detected, is lower than the ratio in previously unexposed persons [8]. These data demonstrate that natural boosting occurred in these mothers.

Meanwhile, we quantitatively measured the measles IgG in ill infants' sera collected between 3-9 days after onset of symptoms (Table 1), and compared the mean titer in infants whose mothers were IgM negative with that in infants whose mothers were IgM positive. Since 14% and 81% of the vaccinees will become positive for measles IgG at week 2 and week 3 respectively after vaccination [10], the measles IgG in the five vaccinated infants might contain the antibody induced by the vaccine. Thus we excluded these five infants in the comparison. The mean measles IgG concentration in infants of the mothers with negative measles IgM was 56 U/ml (43-82 U/ml), whereas that in infants of the mothers with positive measles IgM was 65 U/ml (45-97 U/ml). The mean IgG values in two groups of infants were comparable although the antibodies in their mothers were 10 times different (5450 U/ml vs 57868 U/ml). Additionally, measles IgG in infants No 3 and No 9 (Table 1), whose mothers were both positive for IgM, was far lower than the expected level based on the decay rate with the IgG half-life of 23 days in serum. Therefore, these data provide additional evidence that the high measles IgG in the six mothers was the consequence of recent subclinical infection, rather than the pre-existing measles antibody.

Since the measles IgG detected by ELISA based on the whole virus antigens is not all neutralizing, we analyzed the measles neutralizing antibody by cell culture. Generally, as shown in Table 1, sera that contained high concentrations of measles IgG had high titers of neutralizing activity. According to mothers' status of measles IgM, we compared the measles neutralizing titers in the vaccinated mothers with those in 13 control women with the histories of measles. As shown in Table 2, the mean titer in the vaccinated mothers who had no serological evidence of natural boosting was 37, while that in the 13 control women was 182; the difference was statistically significant (*P *< 0.05). As expected, the mean neutralizing titer in the six vaccinated mothers with recent natural boosting reached to 1109, considerably higher (*P *< 0.05) than either of the two other groups. In an early study reported in 1961, Stokes et al [11] showed that after exposure to wild type virus, half of the naturally immune persons with neutralizing titers from 2 to 8 experienced subclinical reinfections, but none of the individuals with titers from 16 to 128 was subclinically infected. In the present study, of the 18 mothers without the serological evidence of subclinical infections, 14 had neutralizing titers from 16 to 192, two had the titer of 12, and only two had the titers of 4 and 8. Therefore, it is highly possible that neutralizing titers in the 6 mothers positive for measles IgM were < 8 when they labored their infants.

**Table 2:** Measles IgG and neutralizing antibody in mothers and control women (range)

Subject	Number	Mean measles IgG in U/ml	Mean reciprocal neutralizing titer^d^
Control woman^a^	13	9653 (478-70506)	182 (16-1024)
Mother (-) for IgM^b^	18	4740 (275-23078)	37 (4-192)
Mother (+) for IgM^c^	6	57531 (19002-77355)	1109 (256-2048)

^a ^With histories of measles in their childhood.

^b ^Vaccinated without natural boosting.

^c ^Vaccinated with recent natural boosting.

^d ^*P *< 0.05, Kruskal-Wallis rank-sum test.

In this study, we expressed the measles IgG in U/ml according to the reference provided by the ELISA kit manufacturer, rather than in milli-international units per milliliter based on the international standards. Thus, it is difficult to compare our measles IgG levels with other reports and with the conventionally accepted protective level of 200 mIU/ml [12]. However, as reported by others [13], we found that our ELISA measles IgG levels were generally correlated with the neutralizing titers (Table 1). Therefore, the measles IgG detected in this study reflected the real situations and the antibody comparison in the different groups was reliable.

In primary acute measles virus infection, detectable specific IgG antibody generally appears in the serum during late phase of the infection [14]. Thus, the measles IgG in the ill infants within 3-9 days after onset should partially contain, if not all, the maternally derived passive antibody, indicating that maternal measles IgG in these infants was decayed to be under the threshold level for protection because of insufficient antibody acquired from their mothers.

Epidemiologic studies demonstrate that the relative risk of measles among infants whose mothers were born in the vaccine era is greater than that among infants whose mothers were born before availability of the measles vaccine [15]. In the present study, we found that all the 24 mothers had been vaccinated successfully; however, their infants still developed measles before or around the age of measles vaccination. Together with the low measles IgG and neutralizing titers in the mothers when they delivered their infants, our data strongly suggest that infants born to mothers with the vaccine-acquired immunity to measles will have short duration of protection against measles.

Despite the widespread use of measles vaccine and successful controls of the disease globally, resurgence of measles occurred in both developed and developing countries during past decade. In developed countries, relatively low measles vaccination levels among preschool children were attributed to the resurgence; the endemic measles transmission has been interrupted by raising the vaccination coverage in preschool children and performing the catch-up immunization in school children [16,17]. In developing world, however, in addition to suboptimal vaccination coverage, other influential factors may be involved in the resurgence since the high vaccination coverage did not effectively hurdle endemic measles transmission [18,19].

The results in the present study would be meaningful in designing strategy in eliminating measles in developing countries like China. In 2006, China set up the National Plan for Elimination of Measles, aimed at reducing the incidence below one case per one million population and interrupting indigenous transmission of measles by 2012. To achieve this goal, it is required to reach at least 95% vaccine coverage through routine vaccination of infants with the first dose given at 8 months of age and a second dose at 18-24 months of age respectively and to intensify catch-up vaccinations in older children at the entry of kindergarten or elementary school as well as to take other measures. However, the changing age distribution of measles in infants younger than 1 year of age [2], which is at least partially caused by low maternally acquired measles antibody as demonstrated in this study, casts doubt on the effectiveness of current strategy in elimination of measles. The current strategy can reduce the incidence in general population and thereafter diminish exposure opportunities of young infants to measles virus; however, since infants younger than 8 months of age are not eligible for vaccination, many of them will remain to be susceptible to measles virus because of short duration of the immunity conferred by maternal antibody. Susceptible infants in countries with high measles incidences are at considerable risks for the disease. Therefore, in addition to the current strategy, innovative measures to improve the immunity in young infants younger than 8 months of age would be critical to achieve the goal of elimination of measles in unindustrialized countries like China.

## Conclusion

In conclusion, our results suggest that infants born to mothers vaccinated with measles vaccine in their childhood will lose the immunity to measles earlier than those born to mothers with the histories of measles. Although a recent report shows that vaccination at an age of 4.5 months may curtail the measles in younger infants [19], more reports demonstrate that vaccination in younger infants (6-9 months of age) usually has lower seroconversion rates and lower neutralizing titers compared with immunization in old infants (12-15 months of age) [20]. It appears to be logical and more practical to offer an additional measles vaccine in women before pregnancy so that their future infants can get more maternal measles IgG and have longer duration of protection against measles.

## Materials and methods

### Study subjects

Infants ≤ 9 months of age with the clinical diagnosis of measles (fever, maculopapular rash with conjunctivitis, rhinorrhea, or cough), hospitalized at the Nanjing Second Hospital, Nanjing, China, between March 2007 and July 2007, were recruited. All mothers of the ill infants were explained the study and invited to participate. Signed informed consent was obtained from the mothers who agreed and the infants' consent was signed by their mothers. Blood samples were obtained from mothers and infants by venepuncture within 24 hours after the admission. In addition, blood samples from 13 women who had the histories of clinical measles were also included; the samples were collected during summer and fall of 2007. Sera were aliquoted and frozen at -20 °C. The study was approved by the institutional review boards at the Nanjing Second Hospital and the Nanjing Drum Tower Hospital.

### Determination of measles IgM and IgG antibody

Measles IgM was tested by a measles virus IgM ELISA kit (Euroimmun, Medizinische Labordiagnostika AG, Germany). For quantitative determination of measles IgG, serum samples were coded so that the laboratory was unaware of which samples were from mothers and children; however, the sera were arranged so that the samples from an infant and his/her mother were analyzed in parallel. The measurements were performed with a measles virus IgG ELISA kit (IBL, Hamburg, Germany) including the standard measles virus IgG serials of 1, 10, 40, and 250 units per milliliter serum (U/ml). The ELISA is based on the cell culture derived native virus antigens and the cut-off is 10 U/ml. Each sample was diluted appropriately so that the OD450 was located within the ranges of standard serials.

### Measurement of measles neutralizing antibody

Measles neutralizing antibody in paired sera of the ill infant and his/her mother was measured as described elsewhere [21] with some modifications. Briefly, 25 μl of serial two-fold dilutions (1:2 to 1:2048) of heat-inactivated (56 °C for 30 min) serum prepared in DMEM medium was preincubated with an equal volume of Mvi/Jiangsu.PRC/28.05/1 strain of the measles virus containing 100 TCID50 at 37 °C for 3 hour, and then 50 μl of Vero/SLAM cells (10^6 ^cells/ml) was added to the serum-virus mixtures. Two wells were prepared for each dilution and a control for cytotoxicity was included for each serum. To assure constant amount of the live virus used in experiments performed at different times, each test also included virus back titration containing 1000, 100, 10, and 1 TCID_50 _virus. The cells were kept at 37 °C in 5% CO2. On the next day, 100 μl of culture medium was added to each well and the cells were cultured for 7 days. Neutralization titers were expressed as the reciprocal of the highest dilutions that did not induce cytopathic effect. A serum with the neutralization titer of ≥ 1:2 was considered to be positive.

### Statistical Analysis

Analyses were performed with the SPSS Statistical Package version 12.0 (SPSS Inc, Chicago, IL). The Mann-Whitney U and Kruskal-Wallis rank-sum tests were used for comparisons of measles neutralizing antibody titers between groups. A *P *value < 0.05 was considered statistically significant.

## Competing interests

The authors declare that they have no competing interests.

## Authors' contributions

HZ and PSL performed the experiments and analyzed the data and drafted the manuscript and contributed equally to this work. YH and QW collected specimens and assisted in the performance of the experiments. WY diagnosed the patients and collected specimens and clinical data. YHZ designed the study and critically revised the manuscript. All authors read and approved the final manuscript.
